# An immune-related microRNA signature prognostic model for pancreatic carcinoma and association with immune microenvironment

**DOI:** 10.1038/s41598-022-13045-z

**Published:** 2022-09-02

**Authors:** Qian Shen, JunChen Li, Xue Pan, ChuanLong Zhang, XiaoChen Jiang, Yi Li, Yan Chen, Bo Pang

**Affiliations:** 1 Guang’anmen Hospital, China Academy of Chinese Medical Sciences, Beijing, China; 2 Tianjin University of Traditional Chinese Medicine, Tianjin, China; 3 International Medical Department of Guang’anmen Hospital, China Academy of Chinese Medical Sciences, Beijing, China

**Keywords:** Cancer, Immunology

## Abstract

To establish a prognostic model based on immune-related microRNA (miRNA) for pancreatic carcinoma. Weighted correlation network analysis (WGCNA) was performed using the "WGCNA" package to find the key module genes involved in pancreatic carcinoma. Spearman correlation analysis was conducted to screen immune-related miRNAs. Uni- and multi-variate COX regression analyses were carried out to identify miRNAs prognostic for overall survival (OS) of pancreatic carcinoma, which were then combined to generate a prognostic model. Kaplan–Meier survival analysis, receiver operating characteristic (ROC) analysis, distribution plot of survival status in patients and regression analysis were collectively performed to study the accuracy of the model in prognosis. Target genes of the miRNAs in the model were intersected with the key module genes, and a miRNA–mRNA network was generated and visualized by Cytoscape3.8.0. TIMER analysis was conducted to study the abundance of immune infiltrates in tumor microenvironment of pancreatic carcinoma. Expression levels of immune checkpoint genes in subgroups stratified by the model were compared by Wilcoxon test. Gene Set Enrichment Analysis (GSEA) was performed to analyze the enriched signaling pathways between subgroups. Differential analysis revealed 1826 genes differentially up-regulated in pancreatic carcinoma and 1276 genes differentially down-regulated. A total of 700 immune-related miRNAs were obtained, of which 7 miRNAs were significantly associated with OS of patients and used to establish a prognostic model with accurate predictive performance. There were 99 mRNAs overlapped from the 318 target genes of the 7 miRNAs and the key modules genes analyzed by WGCNA. Patient samples were categorized as high or low risk according to the prognostic model, which were significantly associated with dendritic cell infiltration and expression of immune checkpoint genes (TNFSF9, TNFRSF9, KIR3DL1, HAVCR2, CD276 and CD80). GSEA showed remarkably enriched signaling pathways in the two subgroups. This study identified an immune-related 7-miRNA based prognostic model for pancreatic carcinoma, which could be used as a reliable tool for prognosis.

## Introduction

Pancreatic carcinoma (PC) is one of the most fatal malignancies associated with a 5-year survival rate of 8%^1^. First presenting symptoms typically include abdominal or back pain, obstructive jaundice and weight loss. Currently, PC is mainly diagnosed by computed tomography (CT) and treated via surgery. It is poorly sensitive to chemotherapeutic agents. From early asymptomatic in most cases until tumor invasion to surrounding tissues or distance metastasis, approximately 80–85% of the patients have an advanced, un-resectable tumor at the time of diagnosis^2^. In this context, early diagnosis and accurate prognosis are of vital significance for effective management of PC.

MicroRNA (miRNA) is a class of small, non-coding RNA molecules with a size of 17–25 nucleotides^3^. It was first reported by Ambros and Ruvkun early in 1993^4^. It is well believed that miRNA-induced silencing complex (RISC) can bind to the complementary sequences on the 3′-untranslated region of target messenger RNA (mRNA), leading to mRNA decay, translational repression and subsequent regulation of the growth and development of organisms^5^. Some scholars reported suppression of the overall expression of miRNA in tumor cells through 217 mammal samples^6^. miRNAs are important in initiation and progression of tumors by serving as tumor suppressor genes or oncogenes according to their functions^7^. Previous research found that miR-196a and miR-196b were barely expressed in normal tissues but exhibited increased expression in PC tissues. This demonstrated that miR-196a and miR-196b could be diagnostic biomarkers for PC^8^. Another study revealed that the expression levels of miR-574-5p, miR-1244, miR-145, miR-328, miR-26b and miR-4321 were associated with the overall survival (OS) and disease-free survival (DFS) in patients with PC^9^. Based on the wide distribution of miRNA in human plasma, urine, saliva and their stable, non-invasive characteristics, miRNA are promising diagnostic and prognostic biomarkers for tumors.

PC can escape by inhibiting immune checkpoints such as programmed death ligand-1 (PD-L1) and cytotoxic T-lymphocyte-associated protein-4(CTLA-4), which makes immunotherapy become one of the important treatment methods^10^. For PC, immune therapeutic schemes mainly are immune checkpoint inhibitors (ICI), cancer vaccines, adoptive cell transfer (ACT) and combinations with immunotherapeutic agents^11^. It was reported that miRNA could have cancer immunotherapeutic effect by inhibiting immune checkpoints, such as PD-L1^12^. The mechanism of anti-tumor action of miRNA via immunotherapeutic pathways remains to be further explored.

In the current study, transcriptome data of PC were downloaded from The Cancer Genome Atlas (TCGA). Weighted correlation network analysis (WGCNA) was performed using "WGCNA" package to screen key module genes. Immune-related miRNAs were screened out by Spearman correlation analysis and then analyzed by uni- and multi-variate COX regression to obtain immune-related miRNAs prognosis for OS of PC. A prognostic model was constructed based on the identified miRNAs and the performance of the model was validated by Kaplan–Meier (KM) curve, receiver operating characteristic (ROC) curve, uni- and multi-variate COX regression analyses. A miRNA–mRNA interaction network was generated based on the target genes of the model miRNAs and the key module genes. Moreover, gene set enrichment analysis (GSEA), immune cell infiltration in tumor microenvironment (TME) and expression of immune checkpoints were performed and analyzed in subgroups stratified by the model-based risk scoring system. This study aims at providing evidence for further immune therapy and prognosis in patients with PC.

## Methods

### Data collection and processing

**Figure 1:**
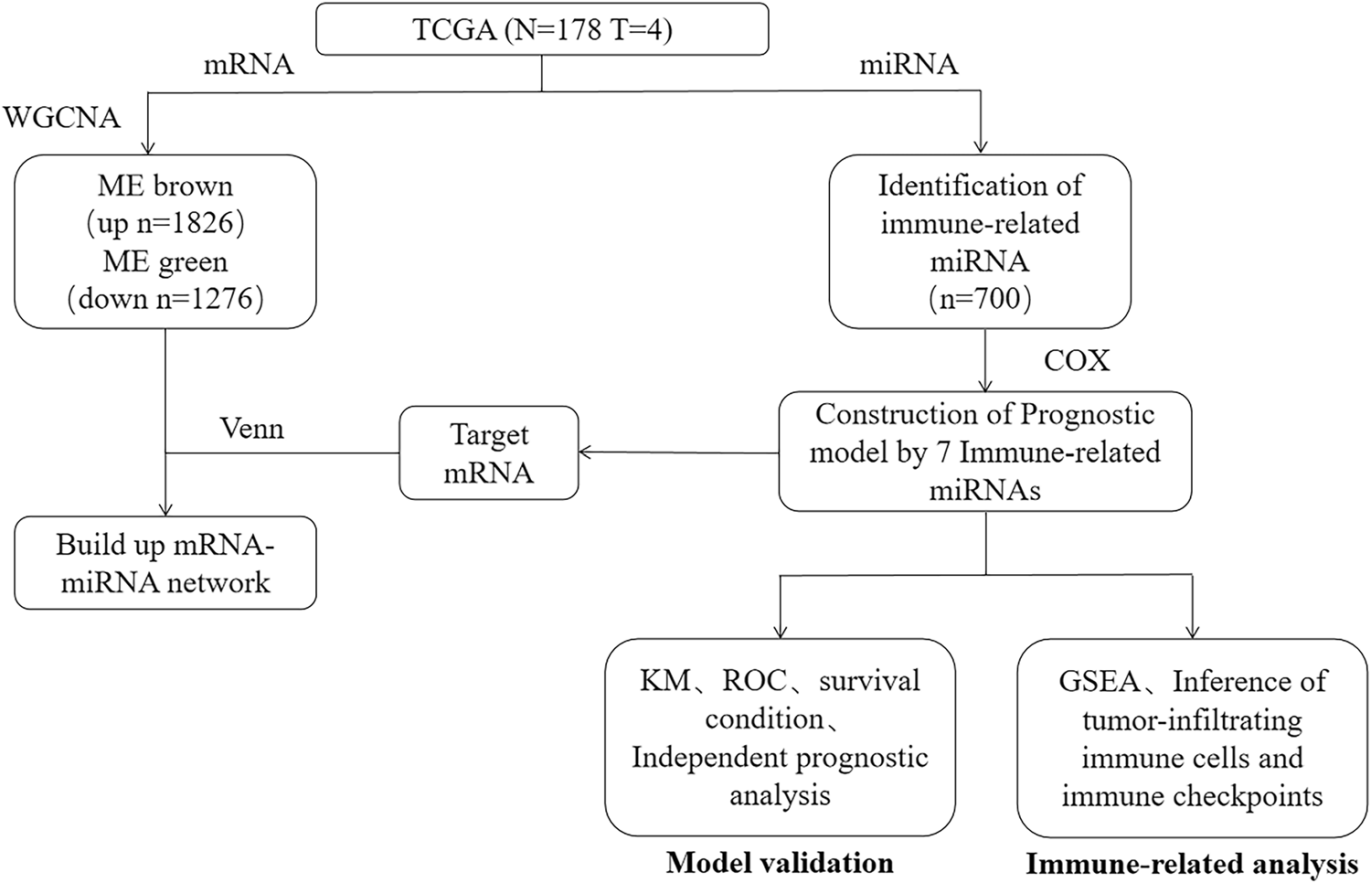
The flow chart.

Expression data of miRNA and mRNA of PC, together with corresponding clinical information, were downloaded from TCGA-PAAD dataset (https://portal.gdc.cancer.gov)^13^ (Fig. 1). According to survival data, 178 tumor samples (mRNA, miRNA) and 4 normal samples (mRNA) were eventually obtained.

### WGCNA analysis for mRNA

R package "WGCNA" was used to construct a co-expression network to find the gene module highly associated with PC^14^. In short, the soft-thresholding power (β) that fitted the criterion of the approximate scale-free topology of the network (R^2^ = 0.9) was determined as 2. Minimum module size was set to 50. An adjacency matrix was generated and then converted to a topological overlap matrix (TOM). Average linkage hierarchical clustering was performed based on the differential TOM measures to classify genes of similar expression patterns into a gene module. *P* value in t test was calculated and visualized.

### Screening of immune-related miRNAs prognosis for PC

Single-sample gene set enrichment analysis (ssGSEA) was performed in samples of the TCGA-PAAD dataset to obtain immune scores of M13664 (immune system process) and M19817 (immune response) gene sets based on the MSigDB database (http://software.broadinstitute.org/gsea/index.jsp)^15–17^. Spearman correlation coefficient was calculated to screen immune score-related miRNA (|R|> 0.3, P < 0.05), which were then intersected with the prognostic miRNAs analyzed by uni-variate analysis to obtain prognostic immune-related miRNAs.

### Construction and validation of immune-related miRNA prognostic model

Immune-related miRNAs prognosis for OS of PC were screened by uni-variate analysis. Further multi-variate COX regression analysis was performed to identify signature miRNAs and construct a miRNA prognostic model(R package "survival")^18^. A risk scoring system was established based on the model and defined as a sum of the product of expression level of each miRNA and corresponding regression coefficient. Each patient was scored and divided into two cohorts by taking the median risk score as the threshold. KM survival analysis was applied to study the prognostic significance of the risk score^19^. ROC curve and distribution plot of survival status in the two cohorts were generated to assess the accuracy of the risk score in prognosis(R package "survivalROC")^20^. Independence of the risk score in prognosis was evaluated using uni- and multi-variate analyses, with the involvement of some other significant clinical features.

### Establishment of immune-related miRNA–mRNA interaction network

Target genes for the model miRNAs were predicted using the miRDB, miRTarBase and Target Scan databases respectively^21–23^. The predicted genes were then intersected with the genes in the key module identified by WGCNA. A miRNA–mRNA interaction network was then generated and visualized by Cytoscape3.8.0. Different regulatory relationships were distinguished by colors^24^.

### Immune cell infiltration and immune checkpoint

CIBERSORT is a deconvolution algorithm developed by Bindea G et al. It can estimate the cell composition and quantify the abundance of specific cell groups in a complex environment using standardized gene expression data^25^. Here, transcriptome data derived from the TCGA-PAAD database were sorted and normalized. Relative abundance of infiltration of 22 immune cells in TME was estimated based on the normalized data using TIMER to study the relationship between the risk score and immune cell infiltration. Since tumor immune therapy has become more popular, expression of immune checkpoint genes in two groups was compared to study the sensitivity of patients to immune therapy.

### GSEA

GSEA was performed using the JAVA GSEA 3.0 (http://software.broadinstitute.org/gsea/index.jsp), by taking the "c2.cp.kegg.v7.4.symbols.gmt" as the reference gene set.

### Statistical analysis

R 4.1.2 and Strawberry Perl fulfilled all statistical analyses of the study. R package "WGCNA" was used to find key module of PC. Spearman correlation analysis was conducted to screen immune-related miRNAs. The model prognostic was established based on multivariate Cox proportional hazards regression model. Both the univariate and the multivariate Cox regression analysis were performed with the “survival” package. The ROC curves were drawn by the package of “survivalROC” in R. Cytoscape3.8.0 software was applied to generate miRNA–mRNA interaction network. TIMER analysis was carried out to study the abundance of immune infiltrates in TME of PC. Expression levels of immune checkpoint genes in subgroups stratified by the model were compared by Wilcoxon test. GSEA was performed to analyze the enriched signaling pathways between subgroups. P < 0.05 was considered statistically significant.

## Results

### Key module identified by WGCNA

**Figure 2:**
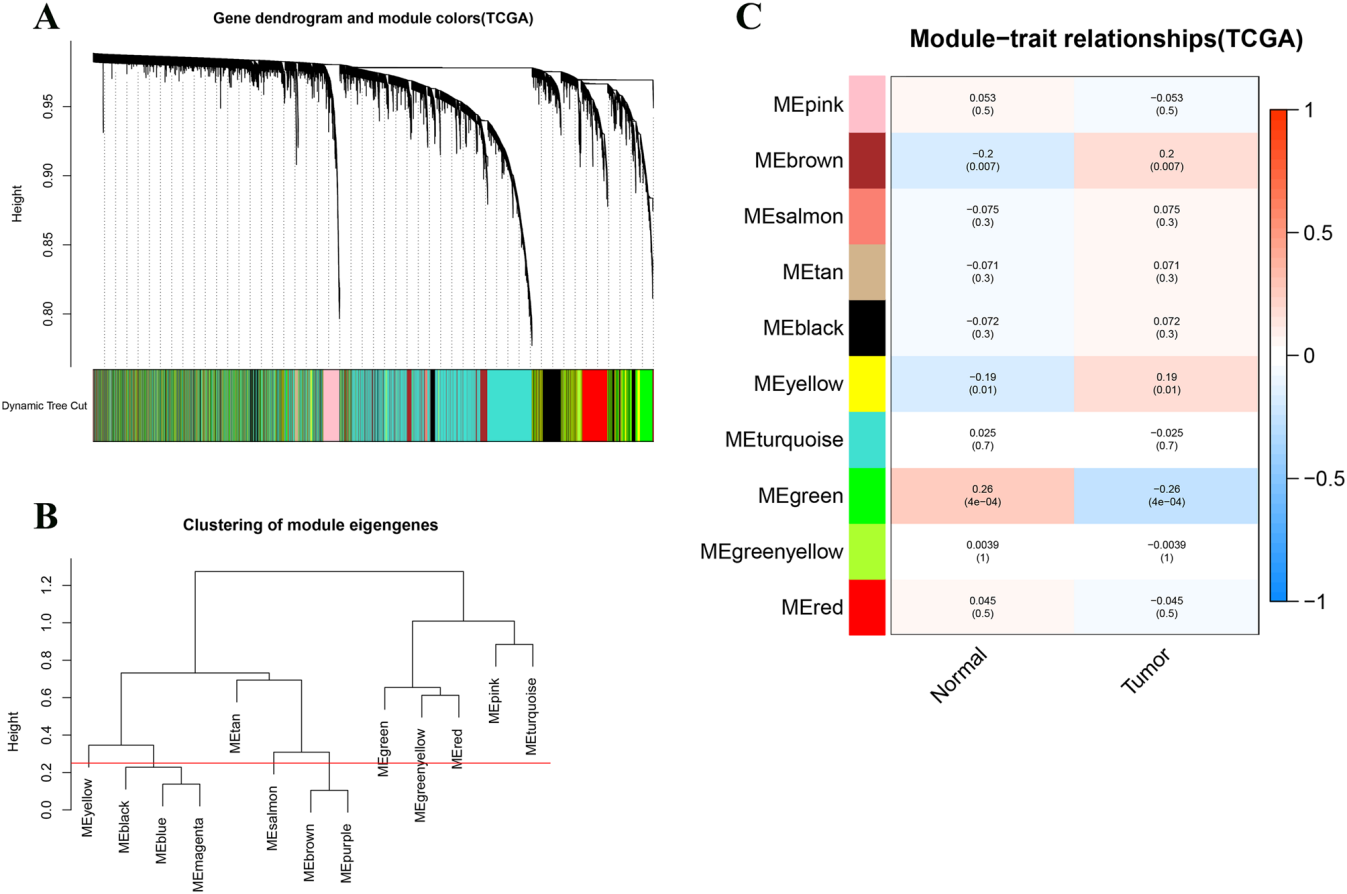
WGCNA identification of cancer-associated mRNA modules in PC. (**A**) Cluster dendrogram of the co-expression network modules created according to the dissimilarity of the topological overlap in the selected mRNAs. (**B**) Shear clustering module. (**C**) Analysis of relationships between genes in modules between PC and normal samples. The green and brown modules were the most tumor-specific modules.

Following WGCNA, 13 gene modules were initially obtained. Further splicing based on Pearson correlation analysis demonstrated 10 gene modules eventually, which were displayed in a Heat map (Fig. 2). The Brown module (r = 0.2, P = 0.07) and Green module (r = − 0.26, P = 4e − 04) were found to be highly associated with PC. Specifically, the Brown module containing 1,826 genes was positively associated with PC, while the Green module composed of 1276 genes was negatively associated with PC. Genes of the two modules were selected for further analysis.

### Screening of immune-related miRNAs

Spearman correlation coefficient was calculated, and a total of 700 immune-related miRNAs were obtained.

### Construction and validation of immune-related miRNA prognostic model

**Figure 3:**
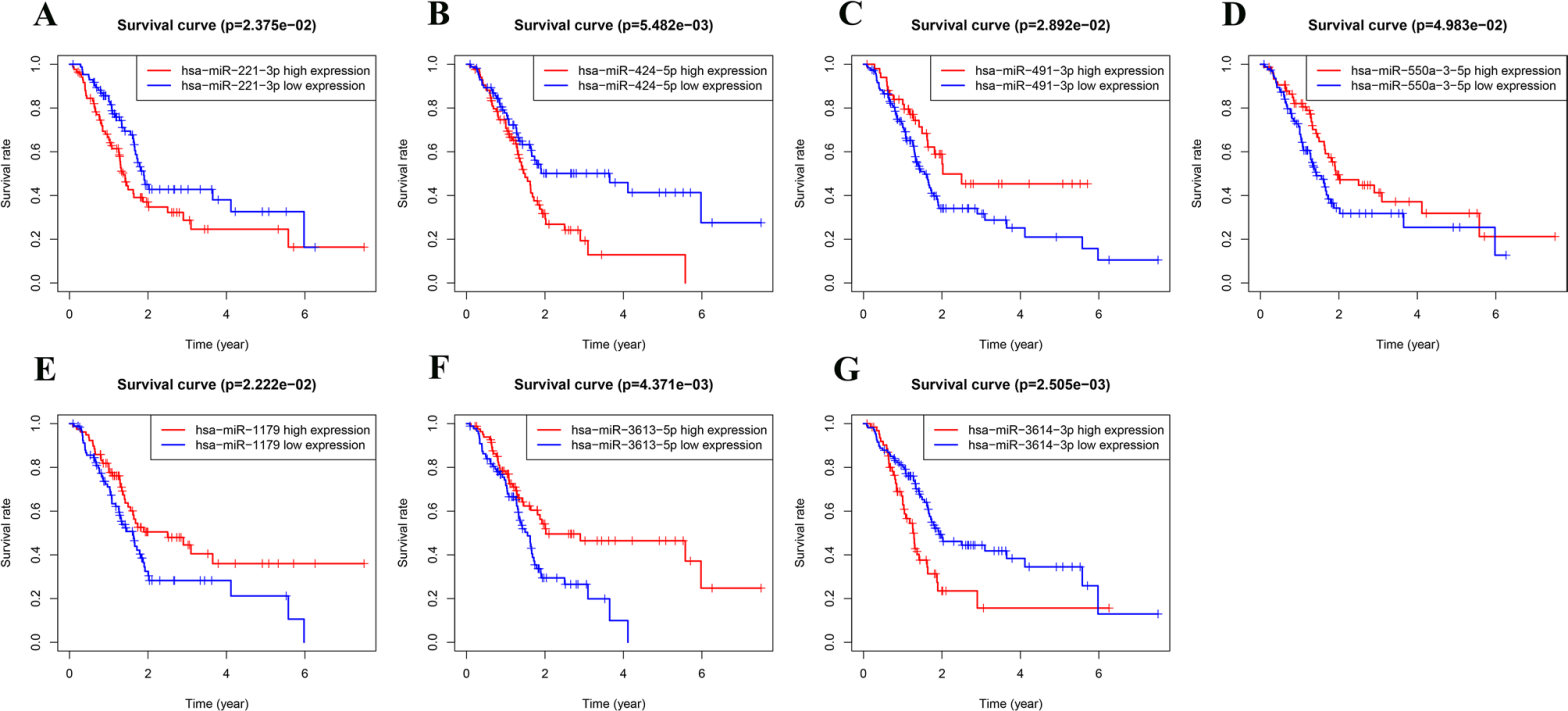
Kaplan–Meier survival curve of 7-immune-related prognostic miRNA. According to the median expression of miRNAs, patients were divided into high-expression groups and low-expression groups. (**A**) KM analysis for patients with hsa-miR-221-3p; (**B**) KM analysi for patients with hsa-miR-424-5p; (**C**) KM analysis for patients with hsa-miR-491-3p; (**D**) KM analysis for patients with hsa-miR-550a-3-5p; (**E**) KM analysis for patients with hsa-miR-1179; (**F**) KM analysis for patients with hsa-miR-3613-5p; (**G**) KM analysis for patients with hsa-miR-3614-3p.

**Figure 4:**
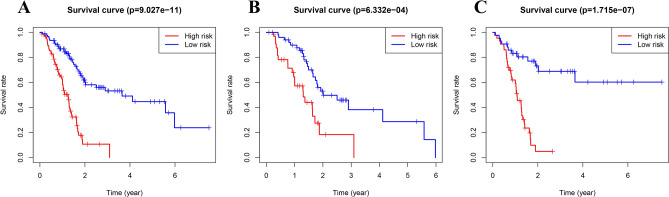
Kaplan–Meier analysis for the 7-immune-related prognostic miRNA signature in PC. (**A**) KM analysis in the the whole set; (**B**) testing; (**C**) training set.

**Figure 5:**
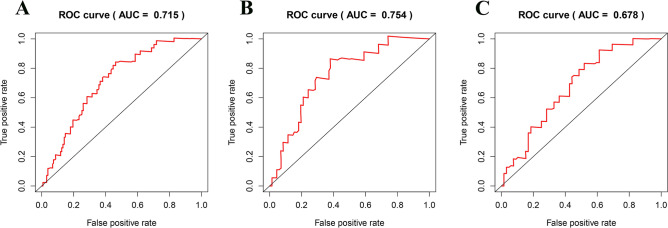
ROC analysis based on time for the 7-immune-related prognostic miRNA signature in PC. (**A**) ROC analysis in the whole set; (**B**) testing set; (**C**) the training set.

**Figure 6:**
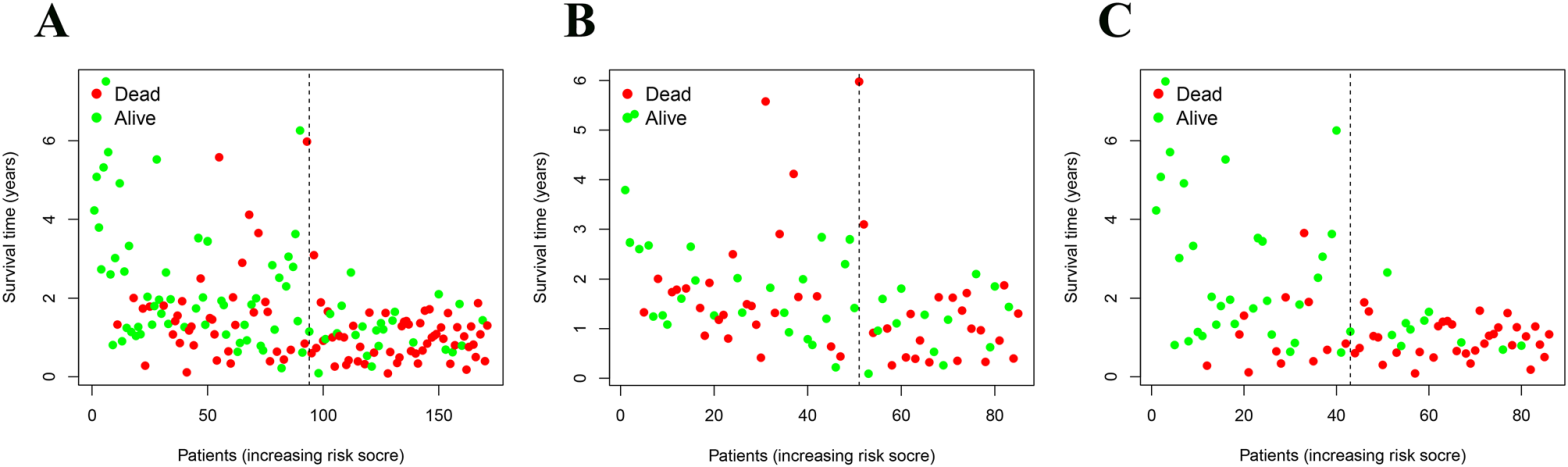
Survival status based on time for the 7-immune-related prognostic miRNA signature in PC. (**A**) survival status analysis in the whole set; (**B**) testing set; (**C**) the training set.

**Figure 7:**
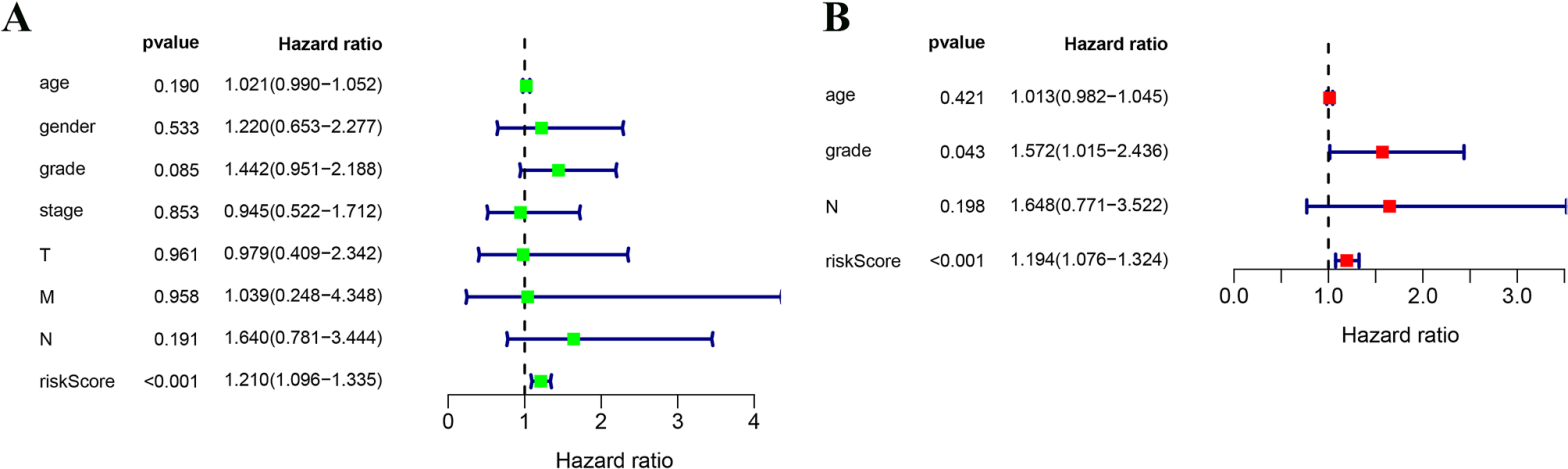
Independent prognostic analysis. (**A**) Forest plot of univariate; (**B**) multivariate.Cox regression analysis showed the risk score was an independent risk factor compared with other clinical features.

700 immune-related miRNAs were randomly divided into train and test sets. Uni-variate COX regression analysis was performed to obtain 20 candidate immune-related miRNAs of prognostic significance in PC (Table 1). Subsequently, multi-variate COX regression models were established and a 7-miRNA based prognostic model was identified (Table 2). A risk score was accordingly established and formulated as miR-550a-3-5p*-0.472400389 + miR-3613-5p*-0.541428145 + miR-221-3p*0.364211355 + miR-424-5p*0.446023047 + miR-491-3p*-0.318762586 + miR-1179*-0.202029941 + miR-3614-3p*0.3625878. Patient samples were divided into two cohorts according to the median risk score. KM survival curves of the 7 model miRNAs were generated. It was found that all these 7 miRNAs were closely associated with the OS of patients, including miR-550a-3-5p, miR-3613-5p, miR-491-3p and miR-1179 showing a negative correlation and miR-221-3p, miR-424-5p and miR-3614-3p showing a positive correlation (Fig. 3). KM survival analysis was also performed in the high- and low-risk cohorts of the train and test sets respectively. Significant difference in OS of the two groups in both sets was observed (P < 0.001) (Fig. 4). AUC values of the ROC curves for survival were 0.715, 0.754 and 0.678 respectively, demonstrating high accuracy of the performance of the model (Fig. 5). Additionally, distribution plot of the survival status of patients in the two groups revealed an increased number of death with the increase of risk score (Fig. 6). Finally, the risk score was detected to be prognosis for OS of PC as detected by uni-variate analysis (*P* < 0.001). According to P < 0.2, further multi-variate analysis indicated that the risk score was independent of other factors in prognosis of PC (*P* < 0.001) (Fig. 7).

**Table 1:** Results of univariate Cox regression analysis of prognostic model.

ID	HR	HR.95L	HR.95H	P value
hsa-miR-885-5p	0.740163659	0.555590702	0.986053656	0.0397893
hsa-miR-1224-3p	0.627159098	0.399845058	0.983702376	0.042202971
hsa-miR-1185-5p	0.675064909	0.492058126	0.92613577	0.014867775
hsa-miR-550a-3-5p	0.678308672	0.477979737	0.962598661	0.02974992
hsa-miR-3613-5p	0.692910252	0.507556443	0.945953152	0.020899474
hsa-miR-577	0.818173964	0.681517027	0.982233179	0.031380084
hsa-miR-221-3p	1.269891406	1.00171102	1.609869664	0.048371667
hsa-miR-1250-5p	0.589350641	0.369257385	0.940628928	0.026653616
hsa-miR-28-3p	1.607754036	1.048731077	2.46476251	0.029388712
hsa-miR-3200-3p	0.769517653	0.61791237	0.958319409	0.019270252
hsa-miR-21-5p	1.593947758	1.130149442	2.24808274	0.007876072
hsa-miR-629-5p	1.633029566	1.090484926	2.445504287	0.017293992
hsa-miR-20b-3p	0.721522995	0.541751777	0.960948268	0.025587082
hsa-miR-424-5p	1.560064118	1.053157977	2.31095439	0.026533694
hsa-miR-203a-3p	1.200146892	1.026659208	1.402951001	0.022007612
hsa-miR-491-3p	0.713184368	0.511413376	0.994561281	0.046359567
hsa-miR-1301-3p	0.711792911	0.529813187	0.956278855	0.024025436
hsa-miR-1179	0.808755735	0.667088946	0.980507687	0.030748622
hsa-miR-7b-5p	1.655289297	1.131640038	2.421249305	0.009395923
hsa-miR-3614-3p	1.62245803	1.215638372	2.165421987	0.001017083

**Table 2:** Results of multivariate Cox regression analysis of prognostic model.

ID	Coefficient	HR	HR.95L	HR.95H	P value
hsa-miR-550a-3-5p	− 0.472400389	0.623503819	0.423264065	0.918473937	0.016836462
hsa-miR-3613-5p	− 0.541428145	0.581916597	0.376964204	0.898299951	0.014520668
hsa-miR-221-3p	0.364211355	1.439378402	1.021559459	2.028085751	0.037352362
hsa-miR-424-5p	0.446023047	1.562087468	0.994572856	2.45343239	0.052826229
hsa-miR-491-3p	− 0.318762586	0.72704814	0.508271886	1.039992597	0.080938806
hsa-miR-1179	− 0.202029941	0.817070464	0.625856531	1.066704763	0.137480041
hsa-miR-3614-3p	0.3625878	1.437043388	1.03999234	1.985681643	0.027975124

### Establishment of immune-related miRNA–mRNA interaction network

**Figure 8:**
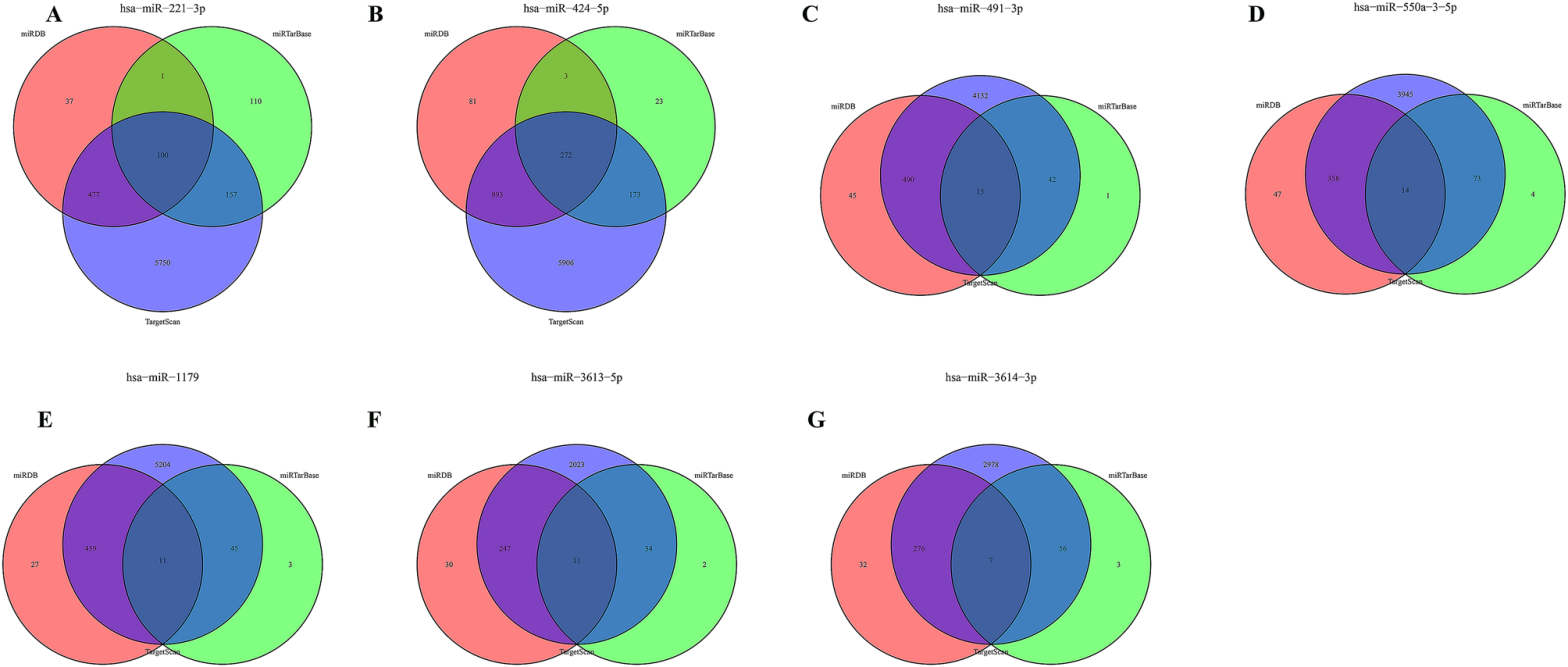
miRNA target Venn. (**A**) Venn diagram of target genes for hsa-miR-221-3p; (**B**) Venn diagram of target genes for hsa-miR-424-5p; (**C**) Venn diagram of target genes for hsa-miR-491-3p; (**D**) Venn diagram of target genes for hsa-miR-550a-3-5p; (**E**) Venn diagram of target genes for hsa-miR-1179; (**F**) Venn diagram of target genes for hsa-miR-3613-5p; (**G**) Venn diagram of target genes for hsa-miR-3614-3p.

**Figure 9:**
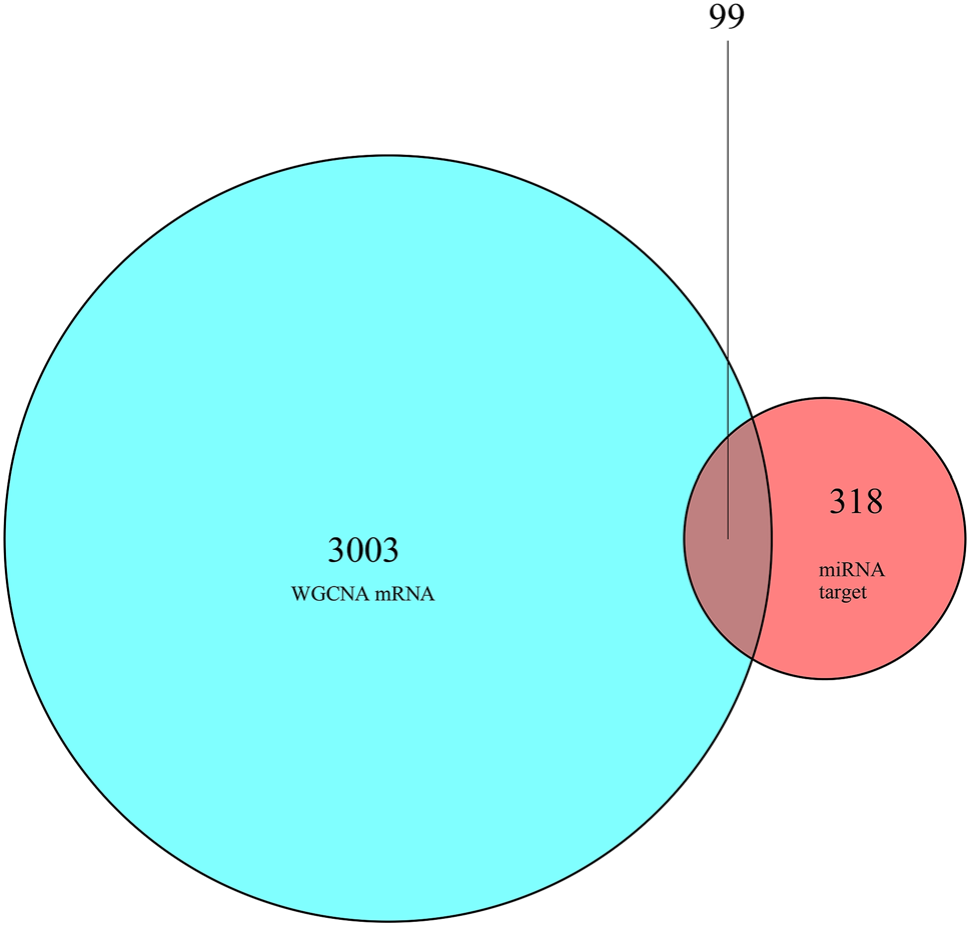
mRNA Venn.

**Figure 10:**
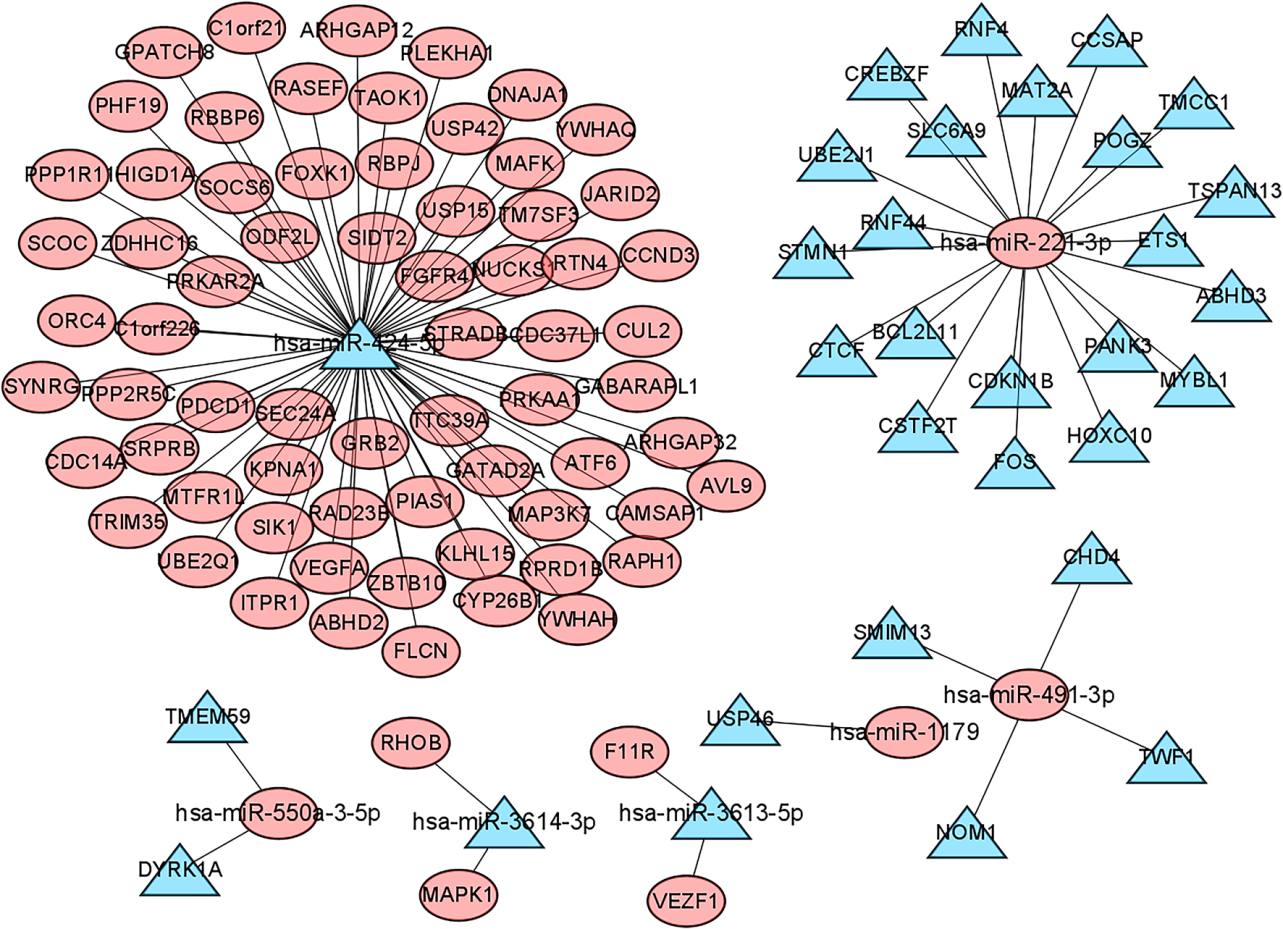
miRNA–mRNA network. Red denoted up-regulated; blue denoted down-regulated.

Target genes for the model miRNAs were predicted using the miRDB, miRTarBase and TargetScan databases respectively. The intersected genes were selected for further analysis, including 100 mRNAs for miR-221-3p, 272 mRNAs for miR-424-5p, 15 mRNAs for miR-491-3p, 14 mRNAs for miR-550a-3-5p, 11 mRNAs for miR-1179, 11 mRNAs for miR-3613-5p, and 7 mRNAs for miR-3614-3p (Fig. 8). After intersection with the key module genes in WGCNA, a total of 99 genes were obtained and considered significantly associated with the immunity and prognosis of PC (Fig. 9). A miRNA–mRNA interaction network was constructed based on the 7 model miRNAs and 99 mRNAs, which the red represented a positive correlation and the blue represented a negative correlation (Fig. 10).

### Immune cell infiltration and immune checkpoint

**Figure 11:**
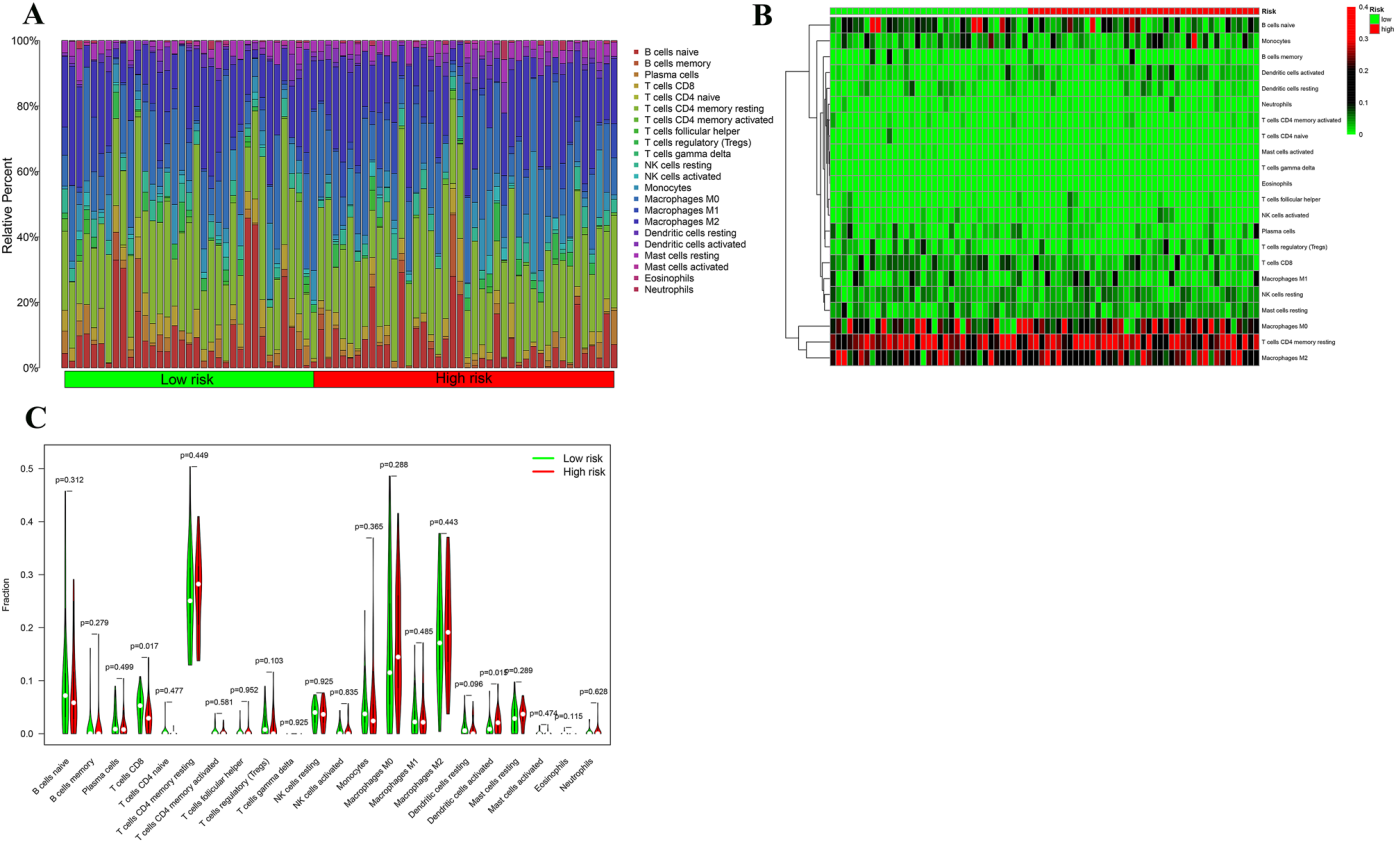
Inference of tumor-infiltrating immune cells. (**A**) CIBERSORT was used to calculate the levels of 22 tumor infiltrating immune cells in PC patients; (**B**) association of risk score and immune cells infiltration; (**C**) differences in the proportion of 22 tumor-infiltrating immune cells between the high and low risk groups (*P < 0.05 **P < 0.01 ***P < 0.001).

**Figure 12:**
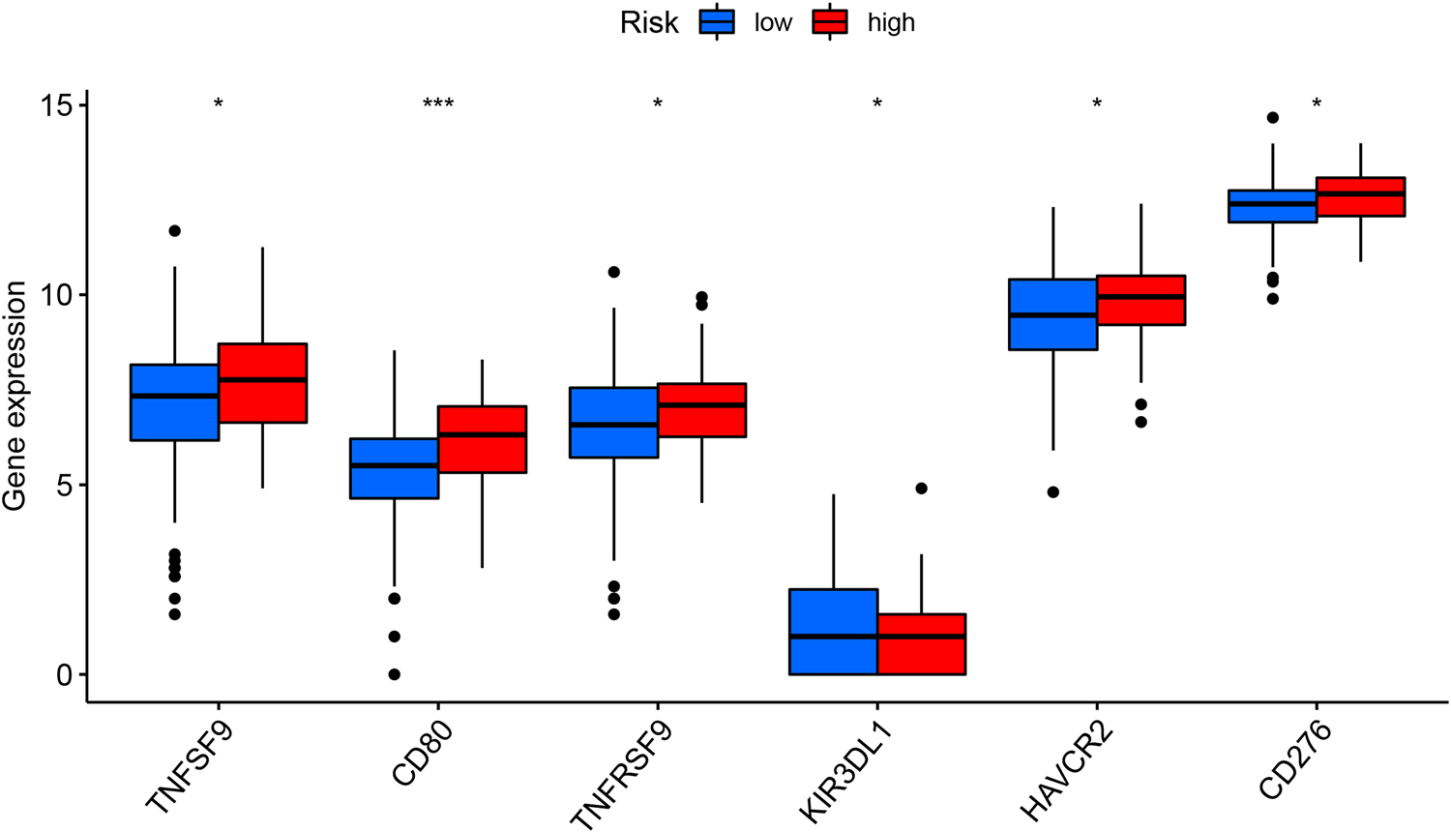
Differences between high and low risk groups in immune checkpoints.

Composition of immune cell subgroups in TME of the high- and low-risk groups was analyzed and the infiltration abundance was displayed by a Heat map (Fig. 11). Infiltration of dendritic cells was demonstrated important between the two groups. Moreover, expression of immune checkpoint genes was examined. It was found that the risk score was associated with expression levels of TNFSF9, TNFRSF9, KIR3DL1, HAVCR2, CD276, especially CD80 (Fig. 12).

### GSEA for target genes of the model miRNAs

**Figure 13:**
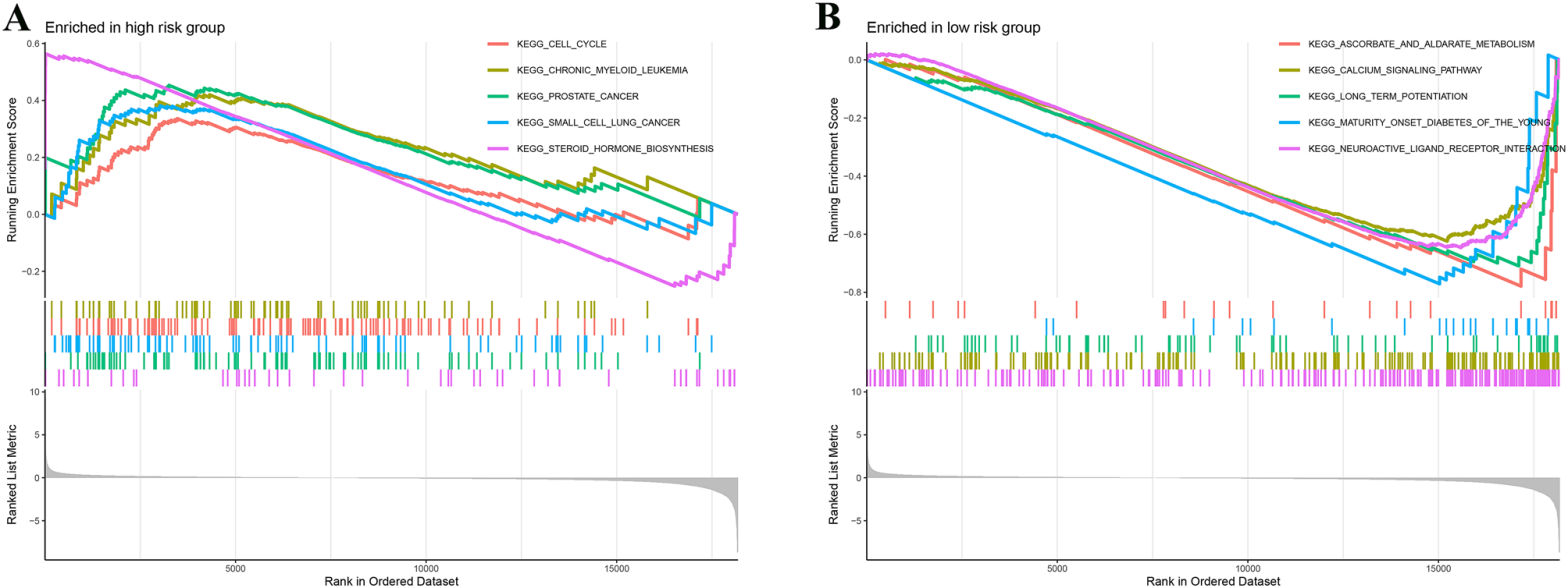
GSEA analysis. (**A**) GSEA analysis of high risk groups; (**B**) GSEA analysis of low risk groups.

By GSEA, the gene set at high risk was mainly involved in the pathways of cell cycle, chronic myeloid leukemia, prostate cancer, small cell lung cancer,steroid hormone biosynthesis. In the meantime, the gene set at low risk was predominantly associated with pathways involved in steroid hormone biosynthesis,calcium signaling pathway,long term potentiation,maturity onset diabetes of the young,neuroactive ligand receptor interaction (Fig. 13).

## Discussion

This study, for the first time, constructed a prognostic model using immune-related miRNAs and validated its accurate, potent performance. We firstly analyzed the key modules associated with prognosis of PC using WGCNA, and then adopted differential analysis to obtain 1826 up-regulated and 1276 down-regulated genes in PC comparing with normal tissues. Spearman correlation coefficient was calculated and 700 immune-related miRNAs were found. Uni- and multi-variate COX regression analyses were performed to screen immune-related miRNA associated with OS. Eventually, a 7-miRNA-based prognostic model for PC was generated and a model-based risk scoring system was established with excellent performance detected by KM, ROC, distribution plot of survival status in subgroups, and uni- and multi-variate COX regression analyses. Furthermore, a miRNA–mRNA network was built using the Cytoscape3.8.0 software, according to the 99 target genes obtained from the predicted mRNAs of the 7 model miRNAs and key module genes. Moreover, immune cell infiltration was analyzed using the TIMER in the subgroups stratified by the model-based risk score, and dendritic cells were found as the most significantly different immune infiltrate in TME of the subgroups. Wilcoxon test also indicated correlation of the risk score with expression of immune checkpoint genes, including TNFSF9, TNFRSF9, KIR3DL1, HAVCR2, CD276, especially CD80. GSEA showed the remarkably enriched signaling pathways in the two subgroups.

There have been multiple studies devoted to constructing prognostic models for PC. For instance, Weng W et al.^26^ established a prognostic model for PC using 14 significant mRNAs; Yan et al.^27^ reported a 4-gene-based prognostic model for PC according to the transcriptome data; Liu et al.^28^ constructed a 5-mRNA prognostic model for PC, which was immune-associated; Chen and Jia^29,30^ obtained a prognostic model based on miRNAs.

Currently, prognostic miRNAs for PC have been extensively studied. Guo et al.^31^ reported that high expression levels of miR-21, miR-212, miR-675, miR-142-5p, miR-204 and low expression levels of miR-148a, miR-187, let-7g were associated with poor prognosis of PC. In that study, they also found several other miRNAs of prognostic significance, including miR-30a-3p, miR-105, miR-127, miR-187, miR-452, miR-518a-2, miR-155, miR-203, miR-210, miR-222, miR-200c, miR-302 and miR-15a. Tesfaye et al.^32^ revealed that the down-regulation of miR-183, miR-34a and the up-regulation of miR-1290, miR-155, miR-203, miR-222, miR-10b predicted a poor survival outcome of PC. Additionally, high expression levels of miR-142-5p, miR-21 led to significantly prolonged survival in patients with PC, and the miRNAs involved in p53, COX2 pathways were demonstrated to be associated with prognosis as well. Gablo et al.^33^ confirmed miR-21 as an oncogene in PC and revealed that increased expression of miR-21 and miR155 was associated with the decline in survival, liver metastasis, lymph node status and increased resistance to Gemcitabine.

In the current study, we successfully established a prognostic model using 7 immune-related miRNAs. Lu et al.^34^ found that expression of miR-550a in severe acute pancreatitis was down-regulated in patients combining with acute lung injury compared with patients without acute lung injury. Qin et al.^35^ revealed that miR-3613-5p was prognostic for renal clear cell carcinoma. Ma et al.^36^ also reported miR-3613-5p as a prognostic biomarker for pancreatic carcinoma and found that the target genes of miR-3613-5p might be correlated with the p53 signaling pathway. Research also found that miR-3613-5p was a key miRNA in cell malignancy of liver cancer induced by aflatoxin B1^37^. Wang et al.^38^ found that miR-221-3p was independent of the prognosis of hepatocellular carcinoma (HCC), while Wang et al.^39^ reported the key role of miR-221-3p in thyroid cancer. Moreover, Fang et al.^40^ established a prognostic model for breast cancer based on 13 miRNAs including miR-221-3p. Abak et al.^41^ analyzed breast biopsy samples and found increased expression of miR-221-3p in tumor tissue compared to that in adjacent normal tissue. Xie et al.^42^ adopted high-throughput sequencing from peripheral blood mononuclear cells in small cell osteosarcoma and detected dysregulation of miR-221-3p. Kandhavelu et al.^43^ found that miR-424-5p was associated with 10 biomarkers of colon carcinoma, while only two of them, including microtubule-associated protein-2 (MAP2) and cyclin gene (CCN) D1, were experimentally validated. Liang et al.^44^ identified miR-424-5p as a potential prognostic biomarker for gastric cancer. In the meantime, Liu et al.^45^ experimentally validated that the up-regulation of miR-424-5p induced by astragaloside IV could inhibit the epithelial-mesenchymal transition (EMT) and angiogenesis in gastric cancer, thereby to play a role in treatment of this cancer. Wang et al.^46^ found and validated the role of miR-424-5p as a factor in prognosis of HCC. Xu et al.^47^ also reported miR-424-5p played a vital role in onset of bile duct carcinoma. Additionally, miR-424-5p might reverse the progression of thyroid cancer via regulating clusterin (CLU) and apolipoprotein (APO)^48^. Ranjha et al.^49^ found decreased expression of miR-491-3p in ulcerative colitis located in the rectal sigmoid colon region than expression in ulcerative colitis located in the ascending colon. circANKRD36 might interact with miR-3614-3p to participate in Type 2 Diabetes and inflammation, supporting their role as potential biomarkers^50^. miR-1179 inhibited the in vivo growth of PC by down-regulating the expression of E2F transcription factor 5^51^. Moreover, miR-1179 could suppress the growth and invasion of thyroid, gastric, esophageal squamous-cell carcinoma, non-small cell lung, cervical, breast, and nasopharyngeal carcinomas via regulating downstream target genes^52–58^.

To conclude, this is the first study that constructed a prognostic model for PC using immune-related miRNAs, aiming to provide a new direction for clinical prognosis of PC. The current study still has some deficiencies, such as lack of validation by molecular biology or relevant clinical trials. Additionally, the miR-491-3p and miR-550a-3-5p have not been fully studied in the field of tumor, requiring further in-depth research.

## Data Availability

TCGA-PAAD datasets is downloaded from the The Cancer Genome Atlas Program (TCGA) database (https://portal.gdc.cancer.gov/projects/TCGA-PAAD). All data and R script in this study are available from the corresponding author on reasonable request.

## References

[1] Siegel RL, Miller KD, Fuchs HE, Jemal A (2021). Cancer statistics, 2021. CA Cancer J. Clin..

[2] Chu LC, Goggins MG, Fishman EK (2017). Diagnosis and detection of pancreatic cancer. Cancer J..

[3] Lee YS, Dutta A (2009). MicroRNAs in cancer. Annu. Rev. Pathol..

[4] Wightman B, Ha I, Ruvkun G (1993). Posttranscriptional regulation of the heterochronic gene lin-14 by lin-4 mediates temporal pattern formation in *C. elegans*. Cell.

[5] Ali Syeda Z, Langden S, Munkhzul C, Lee M, Song SJ (2020). Regulatory mechanism of microRNA expression in cancer. Int. J. Mol. Sci..

[6] Lu J (2005). MicroRNA expression profiles classify human cancers. Nature.

[7] Zhang B, Pan X, Cobb GP, Anderson TA (2007). MicroRNAs as oncogenes and tumor suppressors. Dev. Biol..

[8] Daoud AZ, Mulholland EJ, Cole G, McCarthy HO (2019). MicroRNAs in pancreatic cancer: Biomarkers, prognostic, and therapeutic modulators. BMC Cancer.

[9] Namkung J (2016). Molecular subtypes of pancreatic cancer based on miRNA expression profiles have independent prognostic value. J. Gastroenterol. Hepatol..

[10] Javadrashid D (2021). Pancreatic cancer signaling pathways, genetic alterations, and tumor microenvironment: The barriers affecting the method of treatment. Biomedicines.

[11] Schizas D (2020). Immunotherapy for pancreatic cancer: A 2020 update. Cancer Treat Rev..

[12] Iqbal MA, Arora S, Prakasam G, Calin GA, Syed MA (2019). MicroRNA in lung cancer: Role, mechanisms, pathways and therapeutic relevance. Mol. Aspects Med..

[13] Cerami E (2012). The cBio cancer genomics portal: An open platform for exploring multidimensional cancer genomics data. Cancer Discov..

[14] Langfelder P, Horvath S (2008). WGCNA: An R package for weighted correlation network analysis. BMC Bioinform..

[15] Barbie DA (2009). Systematic RNA interference reveals that oncogenic KRAS-driven cancers require TBK1. Nature.

[16] Wang W (2018). An immune-related lncRNA signature for patients with anaplastic gliomas. J. Neurooncol..

[17] Yoshihara K (2013). Inferring tumour purity and stromal and immune cell admixture from expression data. Nat. Commun..

[18] Stel VS, Dekker FW, Tripepi G, Zoccali C, Jager KJ (2011). Survival analysis II: Cox regression. Nephron. Clin. Pract..

[19] Rizvi AA (2019). gwasurvivr: An R package for genome-wide survival analysis. Bioinformatics.

[20] Heagerty PJ, Lumley T, Pepe MS (2000). Time-dependent ROC curves for censored survival data and a diagnostic marker. Biometrics.

[21] Agarwal V, Bell GW, Nam JW, Bartel DP (2015). Predicting effective microRNA target sites in mammalian mRNAs. Elife.

[22] Wong N, Wang X (2015). miRDB: An online resource for microRNA target prediction and functional annotations. Nucleic Acids Res..

[23] Chou CH (2018). miRTarBase update 2018: A resource for experimentally validated microRNA-target interactions. Nucleic Acids Res..

[24] Shannon P (2003). Cytoscape: A software environment for integrated models of biomolecular interaction networks. Genome Res..

[25] Newman AM (2015). Robust enumeration of cell subsets from tissue expression profiles. Nat. Methods..

[26] Weng W (2020). Identification of a competing endogenous RNA network associated with prognosis of pancreatic adenocarcinoma. Cancer Cell Int..

[27] Yan J (2020). Development of a four-gene prognostic model for pancreatic cancer based on transcriptome dysregulation. Aging.

[28] Liu B, Fu T, He P, Du C, Xu K (2021). Construction of a five-gene prognostic model based on immune-related genes for the prediction of survival in pancreatic cancer. Biosci. Rep..

[29] Jia Y, Shen M, Zhou Y, Liu H (2020). Development of a 12-biomarkers-based prognostic model for pancreatic cancer using multi-omics integrated analysis. Acta Biochim. Pol..

[30] Chen S (2021). Bioinformatics analysis of a prognostic miRNA signature and potential key genes in pancreatic cancer. Front. Oncol..

[31] Guo S, Fesler A, Wang H, Ju J (2018). MicroRNA based prognostic biomarkers in pancreatic Cancer. Biomark. Res..

[32] Tesfaye AA, Azmi AS, Philip PA (2019). miRNA and gene expression in pancreatic ductal adenocarcinoma. Am. J. Pathol..

[33] Gablo NA, Prochazka V, Kala Z, Slaby O, Kiss I (2019). Cell-free microRNAs as non-invasive diagnostic and prognostic bio- markers in pancreatic cancer. Curr. Genom..

[34] Lu XG (2017). Circulating miRNAs as biomarkers for severe acute pancreatitis associated with acute lung injury. World J. Gastroenterol..

[35] Qin S, Shi X, Wang C, Jin P, Ma F (2019). Transcription factor and miRNA interplays can manifest the survival of ccRCC patients. Cancers.

[36] Ma J (2020). Screening potential microRNAs associated with pancreatic cancer: Data mining based on RNA sequencing and microarrays. Exp. Ther. Med..

[37] Zhao J (2022). Gene expression network related to DNA methylation and miRNA regulation during the process of aflatoxin B1-induced malignant transformation of L02 cells. J. Appl. Toxicol..

[38] Wang X (2019). Clustered microRNAs hsa-miR-221-3p/hsa-miR-222-3p and their targeted genes might be prognostic predictors for hepatocellular carcinoma. J. Cancer..

[39] Wang J, Wu L, Jin Y, Li S, Liu X (2020). Identification of key miRNAs in papillary thyroid carcinoma based on data mining and bioinformatics methods. Biomed. Rep..

[40] Fang R (2019). Plasma microRNA pair panels as novel biomarkers for detection of early stage breast cancer. Front. Physiol..

[41] Abak A (2018). Analysis of miRNA-221 expression level in tumors and marginal biopsies from patients with breast cancer (cross-sectional observational study). Clin. Lab..

[42] Xie L (2017). Identification of the miRNA–mRNA regulatory network of small cell osteosarcoma based on RNA-seq. Oncotarget.

[43] Kandhavelu J (2019). Computational analysis of miRNA and their gene targets significantly involved in colorectal cancer progression. Microrna..

[44] Liang Y (2021). MicroRNA profiles in five pairs of early gastric cancer tissues and adjacent non-cancerous tissues. Oncol. Lett..

[45] Liu W, Chen H, Wang D (2021). Protective role of astragaloside IV in gastric cancer through regulation of microRNA-195-5p-mediated PD-L1. Immunopharmacol. Immunotoxicol..

[46] Wang S (2019). A novel multidimensional signature predicts prognosis in hepatocellular carcinoma patients. J. Cell Physiol..

[47] Xu F (2019). Comprehensive analysis of competing endogenous RNA networks associated with cholangiocarcinoma. Exp. Ther. Med..

[48] Nan BY (2021). Comprehensive identification of potential crucial genes and miRNA–mRNA regulatory networks in papillary thyroid cancer. Biomed. Res. Int..

[49] Ranjha R (2015). Site-specific microRNA expression may lead to different subtypes in ulcerative colitis. PLoS ONE.

[50] Fang Y (2018). Screening of circular RNAs and validation of circANKRD36 associated with inflammation in patients with type 2 diabetes mellitus. Int. J. Mol. Med..

[51] Lin C (2018). MicroRNA-1179 inhibits the proliferation, migration and invasion of human pancreatic cancer cells by targeting E2F5. Chem. Biol. Interact..

[52] Yang Y, Ding L, Li Y, Xuan C (2020). Hsa_circ_0039411 promotes tumorigenesis and progression of papillary thyroid cancer by miR-1179/ABCA9 and miR-1205/MTA1 signaling pathways. J. Cell Physiol..

[53] Li Y, Qin C (2019). MiR-1179 inhibits the proliferation of gastric cancer cells by targeting HMGB1. Hum. Cell..

[54] Jiang L (2015). miR-1179 promotes cell invasion through SLIT2/ROBO1 axis in esophageal squamous cell carcinoma. Int. J. Clin. Exp. Pathol..

[55] Song L (2018). MicroRNA-1179 suppresses cell growth and invasion by targeting sperm-associated antigen 5-mediated Akt signaling in human non-small cell lung cancer. Biochem. Biophys. Res. Commun..

[56] Lv F, Zhong Y, Sang L, Wu X (2021). MiR-1179 is downregulated in cervical cancer and its overexpression suppresses cancer cells invasion by targeting CHAF1A/ZEB1. Acta Biochim. Pol..

[57] Li WJ (2018). Increased expression of miR-1179 inhibits breast cancer cell metastasis by modulating Notch signaling pathway and correlates with favorable prognosis. Eur. Rev. Med. Pharmacol. Sci..

[58] Liu D, Wang Y, Zhao Y, Gu X (2020). LncRNA SNHG5 promotes nasopharyngeal carcinoma progression by regulating miR-1179/HMGB3 axis. BMC Cancer.

